# IGF-I induced phosphorylation of PTH receptor enhances osteoblast to osteocyte transition

**DOI:** 10.1038/s41413-017-0002-7

**Published:** 2018-02-26

**Authors:** Tao Qiu, Janet L. Crane, Liang Xie, Lingling Xian, Hui Xie, Xu Cao

**Affiliations:** 1 Department of Orthopaedic Surgery, Johns Hopkins University School of Medicine, Baltimore, MD USA; 2 Department of Pediatrics, Johns Hopkins University School of Medicine, Baltimore, MD USA; 3 State Key Laboratory of Oral Diseases, West China Hospital of Stomatology, Sichuan University, Chengdu, China

## Abstract

Parathyroid hormone (PTH) regulates bone remodeling by activating PTH type 1 receptor (PTH1R) in osteoblasts/osteocytes. Insulin-like growth factor type 1 (IGF-1) stimulates mesenchymal stem cell differentiation to osteoblasts. However, little is known about the signaling mechanisms that regulates the osteoblast-to-osteocyte transition. Here we report that PTH and IGF-I synergistically enhance osteoblast-to-osteocyte differentiation. We identified that a specific tyrosine residue, Y494, on the cytoplasmic domain of PTH1R can be phosphorylated by insulin-like growth factor type I receptor (IGF1R) in vitro. Phosphorylated PTH1R localized to the barbed ends of actin filaments and increased actin polymerization during morphological change of osteoblasts into osteocytes. Disruption of the phosphorylation site reduced actin polymerization and dendrite length. Mouse models with conditional ablation of PTH1R in osteoblasts demonstrated a reduction in the number of osteoctyes and dendrites per osteocyte, with complete overlap of PTH1R with phosphorylated-PTH1R positioning in osteocyte dendrites in wild-type mice. Thus, our findings reveal a novel signaling mechanism that enhances osteoblast-to-osteocyte transition by direct phosphorylation of PTH1R by IGF1R.

## Bone formation: Hormone and growth factor work together

A key hormone and growth factor work together to help turn bone-forming cells into mature bone. Janet Crane and colleagues from Johns Hopkins University School of Medicine in Baltimore, Maryland, USA, tested the effects of parathyroid hormone (PTH) and insulin like-growth factor type 1 (IGF-1) signaling on the differentiation of bone-forming osteoblasts by modulating the activity of their receptors in genetically engineered mice. They found a specific part of the PTH type 1 receptor has a phosphate group added to it by the IGF-1 receptor. This chemical tagging leads to changes in the cytoskeleton of osteoblasts that enhance the formation of mature bone cells known as osteocytes. Mice without this PTH receptor had reduced numbers of osteocytes in their bone. The findings reveal a novel signaling mechanism behind this cellular transition during bone building.

## Introduction

Osteocytes make up over 90% of bone cells and play a major role in control of skeletal tissue homeostasis^1^. Osteocytes regulate bone remodeling, maintain phosphate homeostasis, serve as mechanosensors, and secrete endocrine hormones to communicate with other organs^2^. Osteocytes are terminally differentiated osteoblasts derived from mesenchymal stem cells (MSCs) that become embedded in bone matrix. During the differentiation of osteoblasts to osteocytes, the cells undergo morphological changes, transitioning from a polygonal shape into cells with dendritic extensions^3,4^. While signaling mechanisms that direct differentiation of MSCs to osteoblasts have been extensively studied, the characterization of the transition of osteoblasts to osteocytes is just beginning to be elucidated^2^, but the mechanism regulating changes in cytoskeletal proteins, enzymes, and hormones remains unclear. As osteocytes can survive for up to decades^2^, further studies elucidating factors that influence osteocyte differentiation are essential for understanding disease conditions and therapeutics.

Insulin-like growth factor type 1 (IGF-1) plays a key role in MSC to osteoblast differentiation^5–9^. Osteocytes, similarly to MSCs, osteoprogentior cells, and mature osteoblasts, express IGF-1 and the insulin-like growth factor type 1 receptor (IGF1R)^10^. Disruption of IGF1R in mature osteoblasts and early osteocytes in mice impairs bone formation^11,12^, whereas transgenic overexpression of IGF-1 in mature murine osteoblasts increases osteocyte lacunae occupancy, indicating a potential role in osteoblast-to-osteocyte transition^13^. Clinical and mouse observations suggest an interdependent role of IGF-1 and parathyroid hormone (PTH) for anabolic effects^14^. Both patients who are growth hormone deficient and mice that have been hypophosectomized show a blunted response to PTH, with restoration of the PTH response with co-administration of growth hormone^15–17^. More specifically, global IGF-1 knockout mice and osteoblast-specific IGF1R knockout mice fail to show an anabolic response to PTH in trabecular bone^18–20^. While it is well known that PTH increases IGF-1 mRNA and protein expression^21–25^, Yamaguchi et al. found that downstream IGF-1 signaling events could be detected in response to PTH long before IGF-1 mRNA transcription occurred^26^, suggesting a more complicated relationship between PTH and IGF-1 signaling.

PTH plays a critical role in both osteoblasts and osteocytes by regulating calcium homeostasis and orchestrating bone remodeling^27^. The actions of PTH are mediated by a G-protein-coupled receptor, termed PTH type I receptor (PTH1R)^28,29^, which is expressed in MSCs, osteoblasts, and osteocytes^30,31^. Constitutive activation of PTH1R in murine osteoblasts/osteocytes results in increased trabecular and cortical bone^32,33^, while mice lacking PTH1R in osteoblasts/osteocytes have less trabecular bone formation^34^. PTH1R stimulates downstream signaling events via cyclic AMP production, but can also directly interact with other cell surface proteins to regulate bone remodeling^27^. For example, PTH1R-mediated endocytosis of BMP antagonists and recruitment of low-density lipoprotein-related protein 6 (LRP6) as a co-receptor stabilizes β-catenin and enhances bone morphogenetic protein (BMP) signaling^35,36^. Phosphorylation of PTH1R by transforming growth factor beta 2 receptor leads to endocytosis of both receptors and suppression of TGF-β signaling^37^. In the current study, we investigated if PTH1R and IGF1R directly interact to promote osteoblast-to-osteocyte differentiation. We found in vitro that IGF1R phosphorylated tyrosine 494 (Y494) in the cytoplasmic tail of PTH1R. A novel antibody against this tyrosine-phosphorylation was generated, which facilitated subsequent tracking of phosphorylated-PTH1R in osteoblasts and early osteocytes in vitro and in vivo. We found that the Y494-phosphorylation mediated the preferential targeting of PTH1R to the actin cytoskeleton. PTH signaling enhanced actin filament polymerization to promote outgrowth of osteocyte dendrites. Our findings suggest that a novel PTH-IGF-1 signaling interaction contributes to dendrite outgrowth during osteoblast-to-osteocyte transition.

## Results

### Osteoblast and osteocyte differentiation is enhanced by co-stimulation of IGF-1 and PTH

**Fig. 1:**
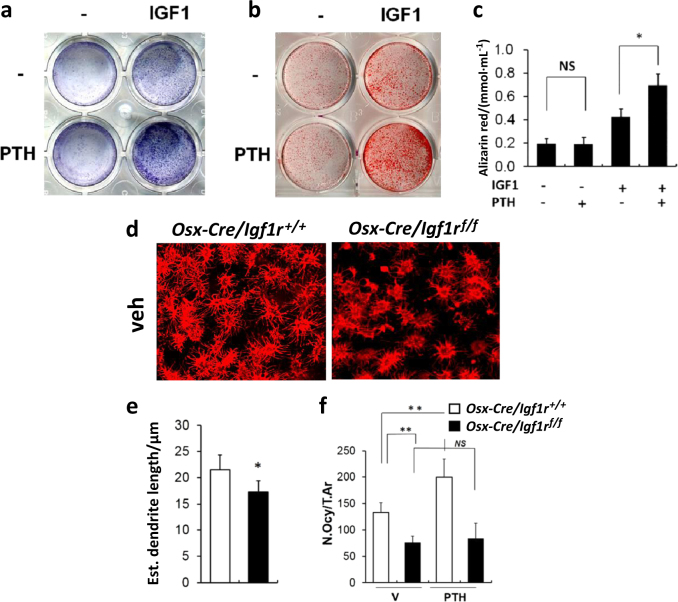
Osteoblast/Osteocyte differentiation is enhanced by co-stimulation of IGF-1 and PTH. **a, b** Osteogenic differentiation of BMSCs treated with vehicle (-), IGF-1 (50 ng·mL^-1^), PTH (100 nmol·L^-1^), or both as assessed by alkaline phosphatase (**a**) and alizarin red staining (**b**). **c** Quantification of alizarin red staining. **d–f** Representative F-actin staining (**d**) and quantification of the number of osteocytes (**e**) and estimated dendritic length (**f**) in calvarial osteocytes in two-month-old Osx-Cre/IGF1R^*f/f*^ mice and wild-type littermates (Osx-Cre/IGF1R^+/+^) treated with either vehicle (veh) or PTH. The data shown as mean ± s.d. *n* = 5. *P < 0.05; **P < 0.01; NS not significant

IGF-1 is known to play a key role in osteoblast differentiation; however, little is known about osteoblast-to-osteocyte differentiation. Alkaline phosphatase (Fig. 1a) and alizarin red staining (Fig. 1b, c) showed that IGF-1 promotes osteoblast differentiation, whereas PTH alone does not. However, PTH and IGF-1 together resulted in increased osteoblast differentiation, suggesting a potential interaction between IGF-1 and PTH signaling (Fig. 1a–c), similar to previous reports^14,20^. To explore if the IGF-1 signaling pathway is important for osteocyte differentiation, we deleted IGF1R selectively in preosteoblasts by crossing floxed IGF1R *(IGF1R*^*f/f*^*)* mice with osterix-Cre (Osx-Cre) mice. Mice lacking IGF1R (Osx-Cre/IGF1R^*f/f*^) had a decreased length of dendrites on osteocytes (Fig. 1d, e) and decreased number of osteocytes per tissue area (Fig. 1f) relative to wild-type littermates (Osx-Cre/IGF1R^*f/f*^). While PTH treatment in wild-type littermates increased the number of osteocytes per tissue area, this effect was lost in the Osx-Cre/IGF1R^*f/f*^ mice (Fig. 1f). These results suggest that both IGF-1 and PTH play a role during osteoblast-to-osteocyte transition, and the role of PTH in osteocytes depends on the presence of IGF1R.

**Fig. 2:**
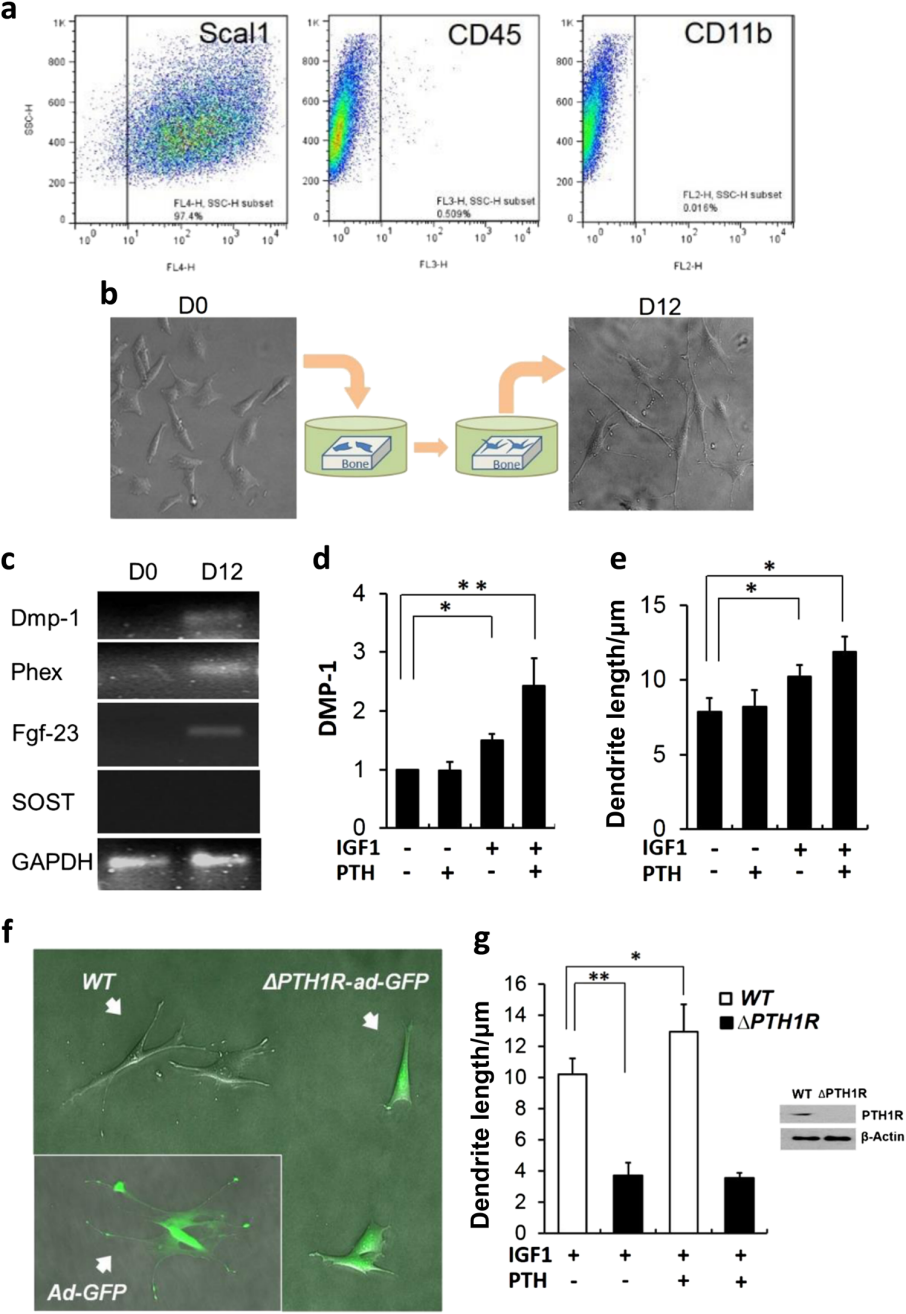
Establishment and validation of IGF-1-PTH effects on an early osteocyte differentiation cell culture. **a** Flow cytometry results of fluorescence-activated cell sorting for Sca1, CD45, and CD11b in primary bone marrow stromal cells (BMSCs). **b** Sca1^+^/CD45^−^/CD11b^−^ BMSCs were seeded on bovine cortical slices in osteogenic medium. Representative photograph of cells at day 0 (D0) and day 12 (D12) of culture. **c** RT-PCR gel of D0 and D12 Sca1^+^/CD45^−^/CD11b^−^ BMSCs for Dmp1, Phex, FGF23, and SOST. **d**, **e** DMP1 expression (**d**) and dendrite length (**e**) of Sca1^+^/CD45^−^/CD11b^−^ BMSCs cultured on bone slices for 12 days and exposed to vehicle, IGF-1 alone, PTH alone, or both IGF-1 and PTH. **f** Representative photograph of Sca1^+^/CD45^−^/CD11b^−^ BMSCs isolated from PTH1R^f/f^ mice and seeded on bovine cortical slices transduced with Adeno-cre-GFP (ΔPTH1R-ad-GFP) or Adeno-GFP as control (PTH1R^+/+^). **g** Dendrite length in PTH1R^f/f^ cells (WT) and Adeno-cre-GFP transfected PTH1R^f/f^ cells (ΔPTH1R) treated with either IGF-1 alone or both IGF-1 and PTH. Inset: Western blot analysis of PTH1R expression in WT and ΔPTH1R cells sorted by GFP expression. The data shown as mean ± s.d. *P < 0.05; **P < 0.01

To further evaluate the role of PTH and IGF-1 in osteoblast-to-osteocyte transition, we developed a cell culture system for early osteocyte-like (EOcyL) cells. We selected primary BMSCs using markers for Sca1, CD45 and CD11b (Fig. 2a). Sca1^+^/CD45^−^/CD11b^−^ BMSCs were seeded on slices of bovine cortical bones in osteogenic medium (Fig. 2b). By 12 days of culture, the morphological appearance of the cells revealed dendrite-like processes (Fig. 2b) and expressed the early osteocyte marker genes Dmp1, Phex and FGF23, but not the mature osteocyte marker SOST (Fig. 2c). EOcyL cells were exposed to PTH alone, IGF-1 alone, or both PTH and IGF-1. DMP1 expression did not change with PTH alone, increased slightly with IGF-1 alone, and increased more significantly with both PTH and IGF-1 relative to vehicle-treated control EOcyL cells (Fig. 2d). A similar pattern of change was noted in regards to dendrite length (2E). We also disrupted the expression of PTH1R in EOcyL cells by isolating and utilizing BMSCs from *PTH1R*^*f/f*^ mice with adenovirus-mediated transduction of Adeno-Cre-GFP. The GFP^+^ transductants were PTH1R-deficient cells (Δ*PTH1R*) and exhibited shortened dendritic processes, relative to negative transductants (GFP^−^) (*PTH1R*^+/+^) (Fig. 2f, g). In contrast, the cells with Adeno-GFP control virus had no such effects (Fig. 2f, inset). While co-treatment with IGF-1 and PTH increased dendrite length in *PTH1R*^+/+^ EOcyL cells, this effect was abrogated in Δ*PTH1R* EOcyL cells (Fig. 2g), again suggesting a relationship between IGF-1 and PTH signaling for osteoblast-to-osteocyte transition.

### PTH1R is directly phosphorylated by IGF1R

**Fig. 3:**
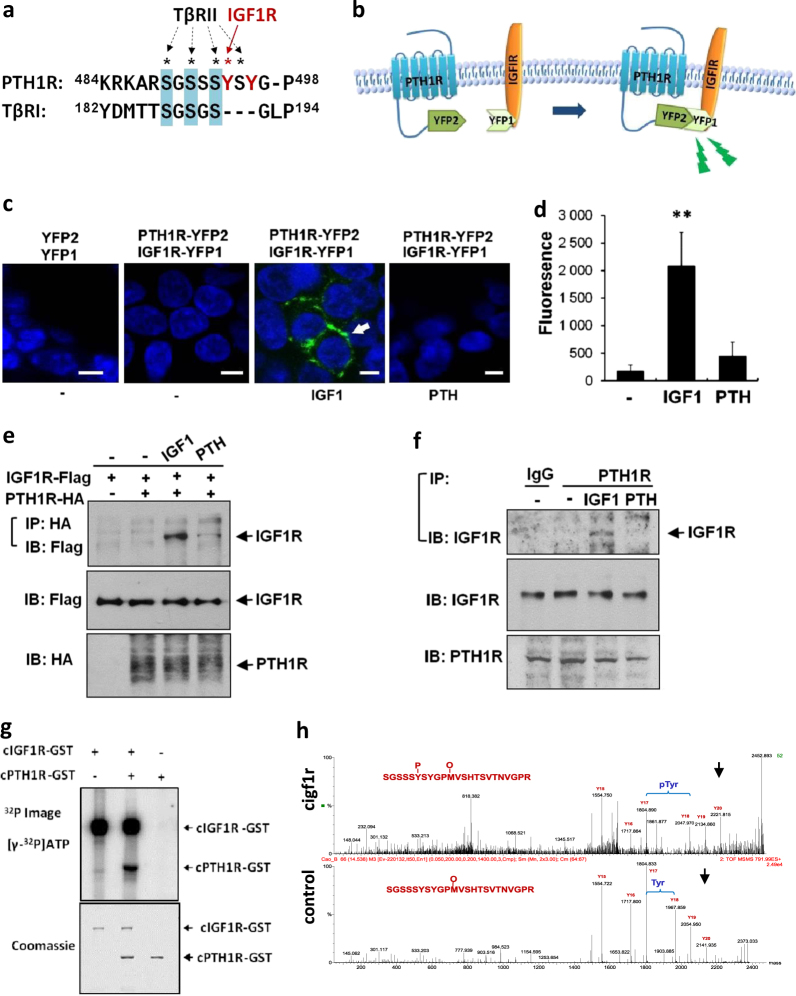
PTH1R is directly phosphorylated by IGF1R. **a** Comparison of amino acid sequence between PTH1R and TβRI. Amino acids 484–498 in the PTH1R cytoplasmic domain share conserved serine residues with TβRI at residues 182–194. The two tyrosines adjacent to the serines in PTH1R are labeled in red. **b** Cartoon representation of strategy of YFP-based protein-fragment complementation assay (YFP-PCA), where immunofluorescence is detected only when YFP1 and YFP2 converge. **c**, **d** YFP-PCA representative images (**c**) and quantitative analysis (**d**) of HEK293 cells transfected with indicated plasmids and then treated with vehicle (−), IGF-1, or PTH for 5 min. Reconstituted YFP fluorescence appears green. DAPI stains nuclei (blue). YFP fluorescence was quantified by fluorimetry and normalized to untransfected cells. Scale bars, 10 μm. **P < 0.01 relative to vehicle. **e**, **f** Immunoprecipitation assays of HEK293 cells transfected with indicated plasmids (**e**) or primary calvarial osteoblasts (**f**). The cells were treated with IGF-1 or PTH for 30 min. Cell extracts were immunoprecipitated (IP) and immunoblotted (IB) with the indicated antibodies. **g** in vitro kinase assay for phosphorylation of IGF1R and PTH1R of native cytoplasmic domain of PTH1R (cPTH1R) alone, IGF1R (cIGF1R) alone, or both. (**h**) Mass spectrometry analysis of cIGF1R phosphorylated peptide vs. unphosphorylated peptide as control. Tyrosines are denoted on the report by Y. No difference on mass unit weight was noted through Y17, whereas an 80.11 g.mol^-1^ difference was noted by Y18, which represents tyrosine 494 [*m/z* 2047.97 in cIGF1R-treated sample vs. *m/z* 1967.86 in control, respectively (arrows)]. Difference in molar mass of phosphotyrosine (261.17 g·mol^-1^) and tyrosine (181.19 g·mol^-1^) is 79.98 g·mol^-1^. HA haemagglutinin

Previously we have identified a serine-enriched region in the C-terminal of PTH1R that could be phosphorylated by TGF-β receptor II (TβRII), a serine/threonine kinase^37^. We therefore questioned if IGF1R, a tyrosine kinase, might directly phosphorylate PTH1R. We noted two tyrosines, directly adjacent to the serines, in the cytoplasmic domain of PTH1R (Fig. 3a). Yellow fluorescent protein (YFP)-based protein-fragment complementation assay (PCA) was utilized in HEK293 cells to investigate whether IGF1R directly interacts with PTH1R (Fig. 3b). Reconstitution of YFP fluorescence from IGF1R-YFP1 and PTH1R-YFP2 was induced on the cell membrane only in the presence of exogenous IGF-1, not with either vehicle or PTH (Fig. 3c, d), indicating that IGF-1 induces a direct interaction between PTH1R and IGF1R. The complex formation of PTH1R with IGF1R was confirmed by immunoprecipitation assays in HEK293 cells transfected with IGF1R-Flag and PTH1R-HA plasmids (Fig. 3e) or primary calvarial osteoblasts (Fig. 3f). The immunoprecipitate was detected only in cells exposed to IGF-1, not vehicle or PTH (Fig. 3e, f). To further determine whether IGF1R phosphorylates PTH1R directly, we prepared a native cytoplasmic domain of PTH1R (cPTH1R)^37^, and tested its potential phosphorylation using the C-terminal of IGF1R (cIGF1R) as a kinase. The cIGF1R exhibited auto-phosphorylation activity and phosphorylated cPTH1R (Fig. 3g). Mass spectrometry analysis of the phosphorylated vs. unphosphorylated peptide identified an 80.11 mass unit shift between the seventeenth and eighteenth tyrosine, corresponding to mono-phosphorylation Y494 of PTH1R (Fig. 3h), indicating that IGF1R kinase directly phosphorylates a specific tyrosine of PTH1R.

### PTH1R and IGF1R interaction does not alter downstream signaling pathways

**Fig. 4:**
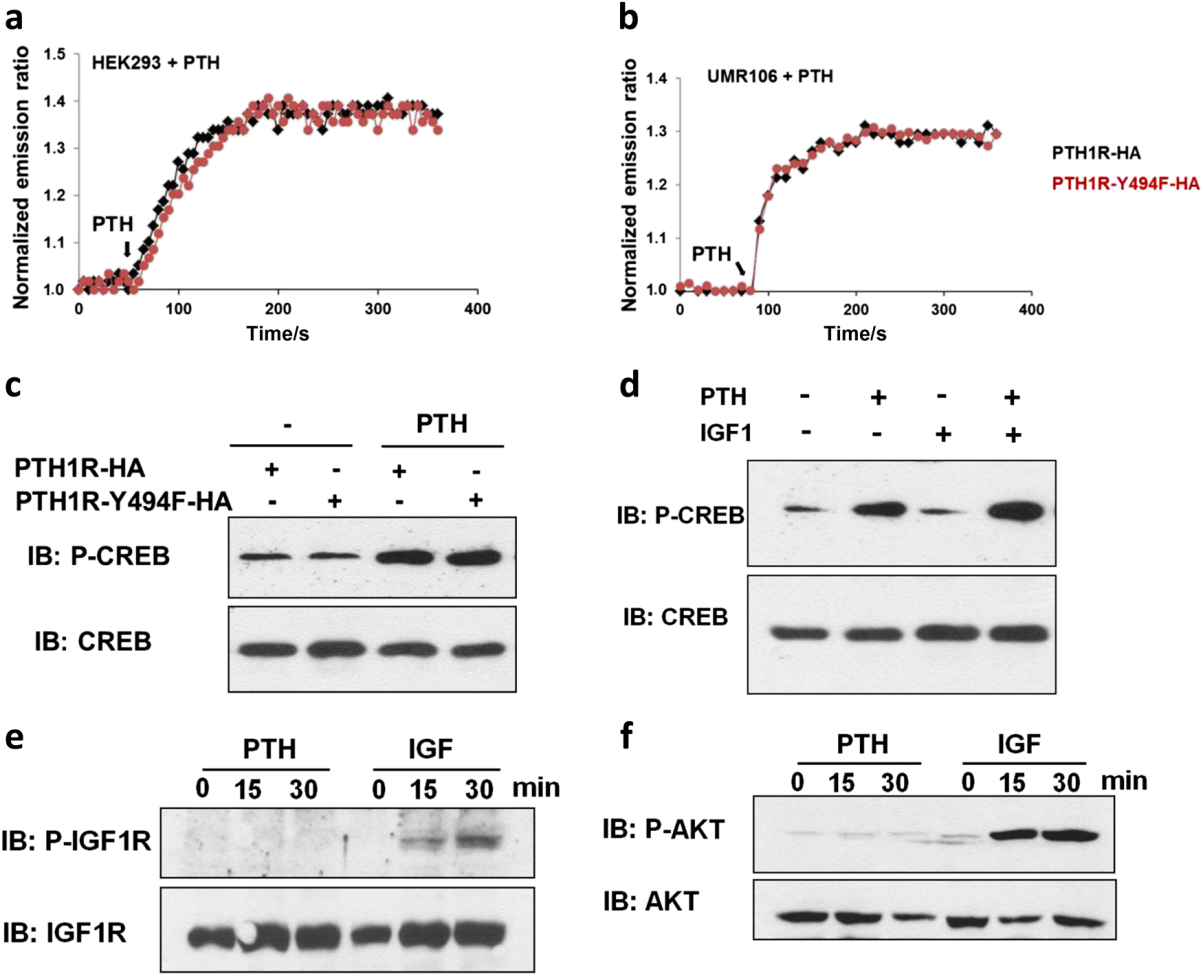
PTH1R and IGF1R interaction does not alter downstream signaling pathways. **a**, **b** HEK293 (**a**) or UMR106 (**b**) cells were co-transfected with ICUE3 and PTH1R-HA or PTH1R-Y494F-HA, and then treated with PTH. Representative emission ratio time courses of ICUE3 are shown (from five experiments). **c** HEK293 cells were transfected with PTH1R-HA or PTH1R-Y494F-HA, and then treated with or without PTH. Immunoblots of phosphorylated or total cAMP response element binding protein (P-CREB and CREB, respectively) are shown. (**d**) Immunoblot of P-CREB and CREB in UMR106 cells treated with PTH and/or IGF-1 as denoted. **e**, **f** Immunoblot for phosphorylation of IGF1R (P-IGF1R) and total IGF1R (**e**) or phosphorylated Akt (P-AKT) and total Akt (**f**) in UMR106 cells treated with either PTH or IGF-1 for 0, 15, and 30 min

To test the effect of Y494-phosphorylation on downstream PTH and IGF-1 signaling, we generated a phenylalanine mutation (pMSCV-PTH1R-Y494F) in PTH1R to make Y494 non-phosphorylatable, while maintaining a similar amino acid structure. We measured the activation of intracellular cAMP using the biosensor ICUE3^38^ in HEK293 and UMR106 cells transfected with wild-type PTH1R or PTH1R-Y494F in response to PTH (Fig. 4a, b). The emission ratio was not altered in PTH1R-Y494F cells relative to control cells (Fig. 4a, b). Phosphorylation of CREB, a cAMP signal transducer, was also similar in cells expressing PTH1R-Y494F compared to those expressing wild-type PTH1R, indicating that PTH/cAMP signaling is independent of Y494-phosphorylation (Fig. 4c). IGF-1 and PTH did not directly cross-activate each other’s downstream signaling targets. Specifically, IGF-1 did not induce the phosphorylation of CREB (Fig. 4d), and PTH did not stimulate the phosphorylation of IGF1R or Akt, downstream components of IGF-1 signaling (Fig. 4e, f).

### IGF-1-induced PTH1R phosphorylation (P_Y_-PTH1R) enhances actin polymerization

**Fig. 5:**
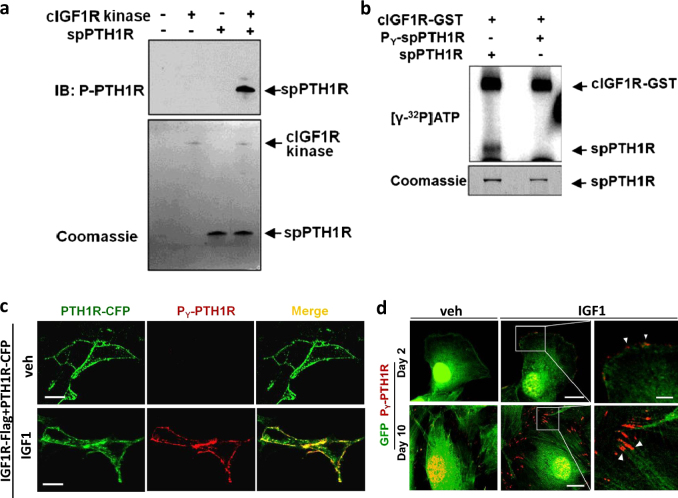
Development and validation of a polyclonal antibody against P_Y_-PTH1R. **a** Kinase assay immunoblot to control, native cytoplasmic domain of IGF1R (cIGF1R) alone, synthesized PTH1R peptide (residues 477–506, spPTH1R) alone, or both cIGF1R and spPTH1R utilizing the synthesized P_Y_-PTH1R antibody (top). Coomassie gel demonstrating appropriate loading of peptides in respective lanes (bottom). **b** Kinase assay immunoblot of a small peptide of PTH1R that was either not phosphorylated (spPTH1R) or phospho-saturated (P_Y_-spPTH1R) at the Y494 position. The cytoplasmic domain of IGF1R (cIGF1R-GST) was only able to induce phosphorylation of spPTH1R, but not P_Y_-spPTH1R. **c** Representative immunofluorescence staining of HEK293 cells stably expressing PTH1R-CFP and transfected IGF1R-Flag treated with vehicle (veh) or IGF-1. Cells immunostained with PTH1R-CFP appear green and synthesized P_Y_-PTH1R antibody are red. Colocalization of PTH1R-CFP and P_Y_-PTH1R appears yellow. Scale bars, 10 μm. **d** Immunofluorescence co-staining of BMSCs expressing GFP (purchased from Texas A&M Health Science Center) plated in osteogenic medium and treated with either vehicle (veh) (left) or IGF-1 (middle) at 2 and 10 days of culture. GFP staining appears green; P_Y_-PTH1R staining appears red. Left panel shows magnification of white boxed area of middle panels. Arrow heads, P_Y_-PTH1R. Scale bars, 20 μm

As the signaling pathways of PTH and IGF-1 were unaffected, we then sought to explore if the phosphorylation of PTH1R by IGF1R played a role in localization of the receptor. We developed and validated a polyclonal antibody against P_Y_-PTH1R to localize and track PTH1R after Y494-phosphorylation. The antibody recognized the antigen only when the cPTH1R peptide was incubated with cIGF1R in a kinase assay (Fig. 5a). To determine the specificity of the PTH1R Y494 phosphorylation, we utilized a small peptide of PTH1R that was either not phosphorylated (spPTH1R) or phospho-saturated (P_Y_-spPTH1R) at the Y494 position. cIGF1R was only able to induce phosphorylation of spPTH1R, but not P_Y_-spPTH1R (Fig. 5b). We further tested the P_Y_-PTH1R antibody in HEK293 cells co-expressing IGF1R and PTH1R fused with CFP (PTH1R-CFP). PTH1R was detected on the cell membrane in vehicle and IGF-1 exposed cells, whereas P_Y_-PTH1R was observed on the cell membrane only after exposure to IGF-1 (Fig. 5c). BMSCs expressing GFP and plated in osteogenic medium for EOcyL cell culture were supplemented with vehicle or IGF-1. P_Y_-PTH1R signals were rarely detected in BMSCs treated with vehicle (Fig. 5d, left panels). In the IGF-1 supplemented BMSCs in EOcyL cell culture system, by day 2 of culture, P_Y_-PTH1R was observed on the cell surface (Fig. 5d, upper panels) and by day 10 of culture, P_Y_-PTH1R was observed on the tips of the dendritic-like processes (Fig. 5d, lower panels).

**Fig. 6:**
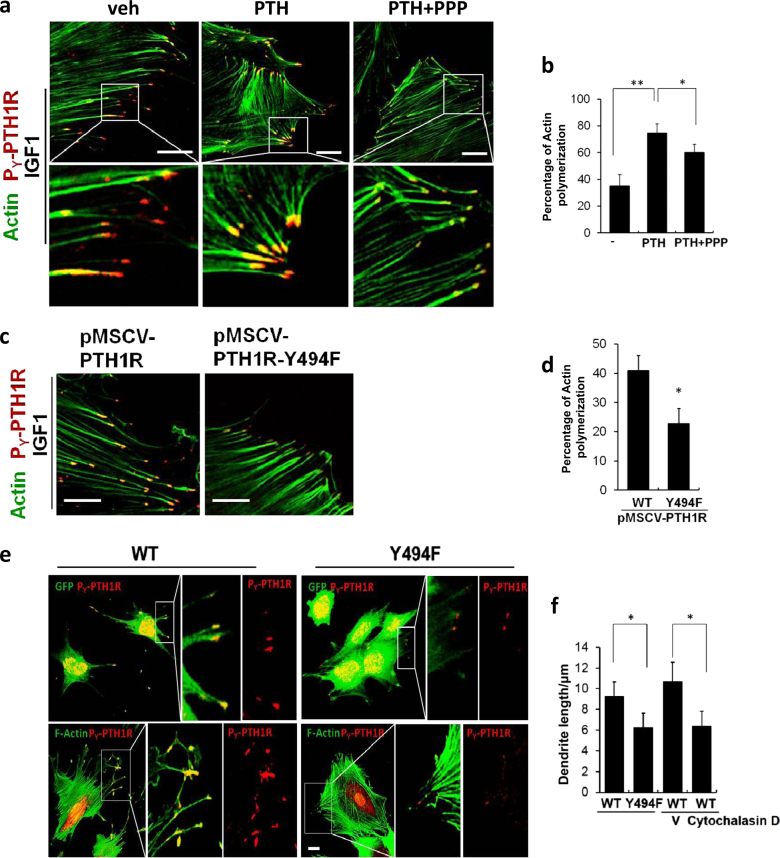
IGF-1 mediated-PTH1R phosphorylation (P_Y_-PTH1R) enhances actin polymerization. **a**, **b** Immunofluorescence staining for actin (green) and P_Y_-PTH1R (red) (**a**) and quantification of actin polymerization (**b**) of BMSCs isolated from 4-week-old mice and cultured in osteogenic medium for 10 days treated with either vehicle (veh), PTH alone, or PTH plus cyclolignan picropodophyllin. Scale bars, 20 μm. The data shown as mean ± s.d. *P < 0.05; **P < 0.01. **c** Immunofluorescence staining for actin (green) and P_Y_-PTH1R (red) (**c**) and quantification of actin polymerization (**d**) of HEK293 cells transfected with murine stem cell virus plasmid and wild-type PTH1R-CFP (pMSCV-PTH1R) or PTH1R-CFP mutant resulting in change of the tyrosine residue at amino acid 494 to phenylalanine (pMSCV-PTH1R-Y494F) and treated with IGF-1. Scale bars, 20 μm. The data shown as mean ± s.d. *P < 0.05; **P < 0.01. **e** Immunofluorescence staining for phalloidin-FITC-GFP (green) (top panels), F-actin (green) (bottom panels) and P_Y_-PTH1R (red) in pMSCV-PTH1R (WT) and pMSCV-PTH1R-Y494F (Y494F) Sca1^+^/CD45^−^/CD11b^−^ BMSCs cultured on bone slices for 12 days (early osteocyle culture media protocol) (EOcyl). Middle (GFP or F-actin staining alone) and right (P_Y_-PTH1R staining alone) panels show magnification of white boxed area of left panel. Scale bars, 20 μm. (**f**) Quantification of dendrite length in pMSCV-PTH1R (WT) and pMSCV-PTH1R-Y494F (Y494F) EOcyl cells without additives to media or treated with vehicle (V) or the actin polymerization inhibitor, cytochalasin D. The data shown as mean ± s.d. *P < 0.05; **P < 0.01

Barbed ends of polymerizing actin filaments push the membrane and promote the formation of plasma membrane protrusions or invaginations^22–24^. Co-staining of P_Y_-PTH1R and F-actin revealed that P_Y_-PTH1R was trans-localized to the barbed ends of F-actin filaments of BMSCs and treated with IGF-1 (Fig. 6a, left panels). Treatment with PTH increased actin polymerization, whereas PTH plus cyclolignan picropodophyllin (PPP), an IGF1R kinase inhibitor, blunted actin polymerization (Fig. 6a, b). P_Y_-PTH1R staining and actin polymerization were also both decreased in pMSCV-PTH1R-Y494F transfected HEK293 cells relative to wild-type control (pMSCV-PTH1R) (Fig. 6c, d), indicating that the Y494-phosphorylation site specifically contributed to polymerization of actin filaments. Finally, we transfected Sca1^+^/CD45^−^/CD11b^−^ BMSCs with either pMSCV-PTH1R-Y494F or pMSCV-PTH1R and subjected the cells to EOcyL conditions. Mineralization as assessed by alizarin red was reduced, whereas cell viability was not affected (Supplementary Fig. S1A, B). As expected, disruption of the Y494-phosphorylation site resulted in a decrease in P_Y_-PTH1R signal relative to wild-type control cells (pMSCV-PTH1R BMSCs) (Fig. 6e). The length of dendrites was decreased in Y494F cells relative to wild-type controls (Fig. 6e, f). Inhibition of the actin bundling using cytochalasin D, an actin polymerization inhibitor in wild-type controls also had a decreased dendrite length relative to vehicle-treated wild-type control cells (Fig.6f, right two). These results suggest that P_Y_-PTH1R contributes to the outgrowth of dendrites during the transition of osteoblasts into early osteocytes.

### P_Y_-PTH1R localizes to dendrites during osteoblast-to-osteocyte transition in vivo

**Fig. 7:**
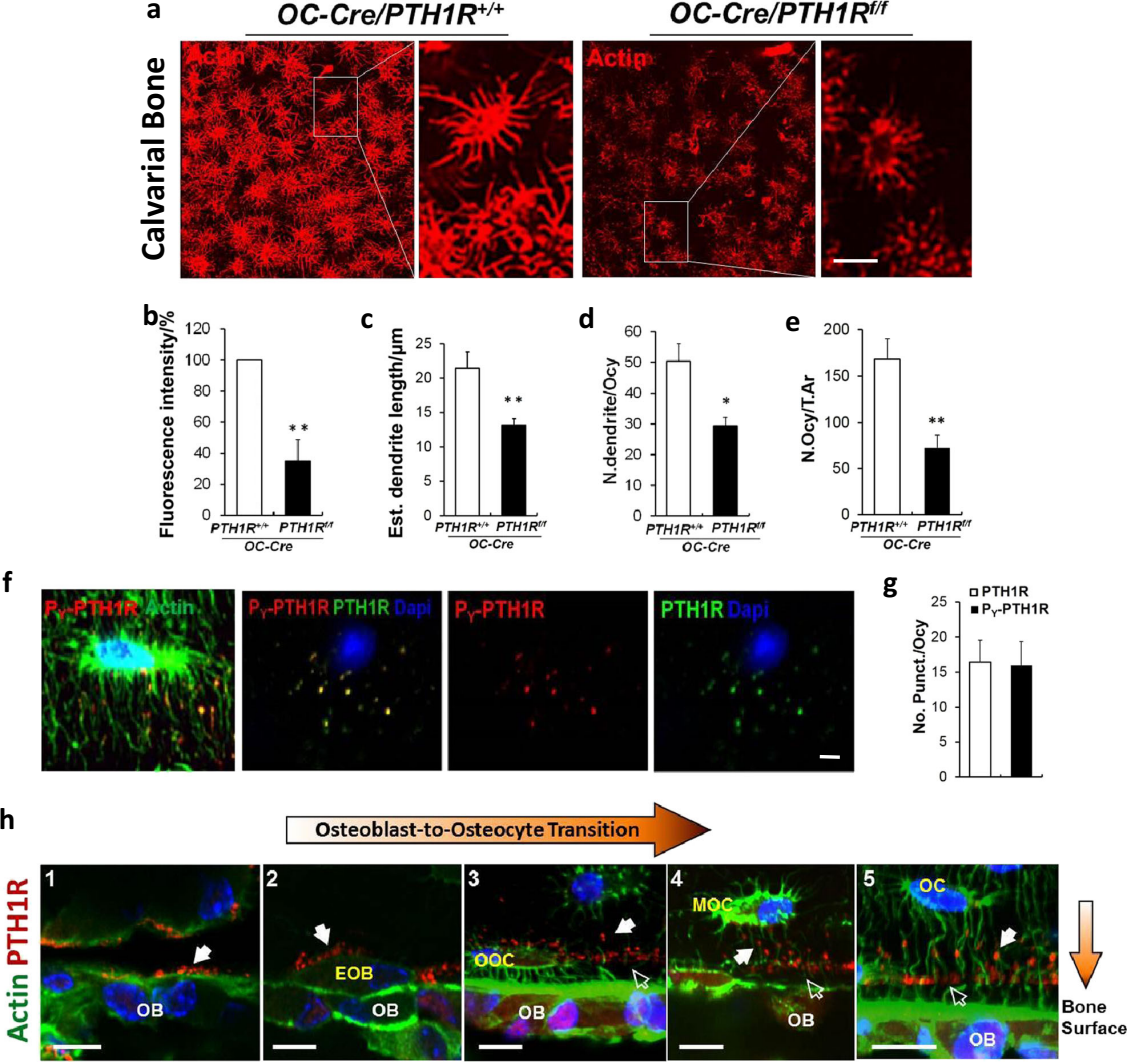
P_Y_-PTH1R localizes to dendrites during osteoblast-to-osteocyte differentiation in vivo. **a** Immunofluorescence staining of osteocytes with phalloidin-Texas Red for F-actin (red) in calvaria of 4-week-old mice with targeted deletion of PTH1R in cells expressing osteocalcin (*Oc-Cre/PTH1R*^*f/f*^) and their wild-type littermates (*Oc-Cre/PTH1R*^+/+^*)*. Left panel shows magnification of white boxed area of right panels. **b**–**e** Quantification of the osteocyte phalloidin-Texas Red fluorescence intensity of actin expressed as percent of *OC-Cre/PTH1R*^+/+^ (**b**), dendrite length (**c**), number of dendrites per osteocyte (*N*. dendrites/Ocy) (**d**), number of osteoctyes per tissue area (*N*. Ocy/T.Ar) (**e**), in *Oc-Cre/PTH1R*^+/+^ and *Oc-Cre/PTH1R*^*f/f*^ calvaria (**b**–**d**) or femur (**e**). *n* = 5. (**f**) Immunofluorescence staining of P_Y_-PTH1R (red) and F-Actin (green) (1st panel), and colocalization (yellow—2nd panel) of P_Y_-PTH1R (red—3rd panel) and PTH1R (green—4th panel) on extending dendrites in 4-week-old wild-type endosteal cortical bone. (**g**) Quantitative analysis of P_Y_-PTH1R and PTH1R punctuates per osteocyte (No. Punct./Ocy). *n* = 5. **h** Immunofluorescence of femur distal metaphyseal trabecular bone in 6-week-old wild-type mice using PTH1R antibody (red) and Actin phalloidin-FITC (green). Localization of PTH1R (red) in osteoblasts (Ob—1st panel), embedding osteoblasts (EOb—2nd panel), osteoid osteocytes (OOcy—3rd panel), mineralizing osteocytes (MOcy—4th panel) and osteocytes (Ocy—5th panel) are denoted by solid white arrows. Dapi stains nuclei (blue). Empty white arrows denoted PTH1R near bone surface. The data shown as mean ± s.d. *P < 0.05; **P < 0.01. Scale bars, 10 μm

To understand the in vivo role of PTH1R on osteoblast-to-osteocyte transition, we analyzed calvarial and cortical bone in mice with Cre-mediated loss of *PTH1R* driven by the osteocalcin promoter (*OC-Cre/PTH1R*^*f/f*^) relative to wild-type littermates (*OC-Cre/PTH1R*^+/+^). In calvarial bone, *OC-Cre/PTH1R*^*f/f*^ mice had less intense fluorescence staining, decreased dendrite length, and decreased number of dendrites per osteocyte relative to *OC-Cre/PTH1R*^+/+^ mice (Fig. 7a–d). In the femur, the number of osteocytes in femoral bone was significantly decreased in *OC*-*Cre/PTH1R*^*f/f*^ mice relative to *Oc-Cre/PTH1R*^+/+^ mice (Fig. 7e). P_Y_-PTH1R was localized to the dendrites in wild-type femoral endocortical bone (Fig. 7f, left panel). Furthermore, PTH1R and P_Y_-PTH1R co-localized in osteocytes (Fig. 7f, right 3 panels) and occurred at similar frequencies (Fig. 7g). Immunofluorescence of PTH1R in femoral metaphyseal trabecular bones of osteocytes at various stages of maturity, as defined by phenotypic appearance previously described^3^, revealed positive staining on the side of osteoblasts (OB) along the bone surface (Fig. 7h, panel 1). The dendritic cytoskeletons of embedding osteoblasts (EOB) and osteoid osteocytes (OOC) also revealed positive staining for PTH1R towards the bone surface (Fig. 7h, panels 2 and 3). In mineralizing osteocytes (MOC), PTH1R was noted mainly on the end of dendrites and occasionally on dendrites closer to the osteocyte cell body projecting towards the bone surface (Fig. 7h, panels 4 and 5).

## Discussion

Osteocytes are dispersed throughout the mineralized matrix, communicating with other osteocytes and cells in the bone marrow via dendritic processes. Extension of dendritic process is thought to be key to the osteocytes role in bone remodeling and mechanosensory signaling. Factors associated with osteocyte dendrite growth, the major morphological change noted in the transition of osteoblasts to osteocytes are under investigation. Podoplanin/gp38 expression has been noted in embedded osteoid osteocytes^39,40^. While fluid-flow shear stress increases the number and length of dendrites, blockade of podoplanin/gp38 blocks this response. Other important factors involved in eliciting and maintaining osteocyte dendrites include cytoskeleton proteins (i.e., tubulin, vimentin, actin, CapG, and destrin)^41–43^, enzymes, and hormones. Specifically, during the transition of osteoblasts to osteocytes, alkaline phosphatase expression is reduced while casein kinase II and osteocalcin are increased^44^. In the current study, we found that PTH1R can be phosphorylated by IGF1R. The phosphorylation mediated preferential targeting of PTH1R to the cytoskeleton, specifically, the barbed ends of actin filaments. PTH-stimulated polymerization of these actin filaments at the barbed ends and promoted the dendrite outgrowth of osteoblasts. Disruption of the phosphorylation inhibited the cytoskeletal targeting of PTH1R and led to decreased dendrite length.

PTH regulates bone remodeling by orchestrating the signaling of local factors, such as TGF-β, Wnts, bone morphogenetic protein (BMP), and IGF-1^27^. PTH elicits these effects via cell membrane receptor interactions, stabilization of downstream signaling events, and gene transcription^21-25, 35-37^. Our group and others have previously reported that serine residues on the PTH1R cytoplasmic tail can be phosphorylated^37, 45-48^. The serine-phosphorylation promotes the internalization and desensitization responses of PTH1R^49,50^ and regulates cAMP, calcium and phosphate homeostasis^37, 51,52^. In the current study, we noted two tyrosines in close proximity to the serine residues. We found that a specific tyrosine residue, Y494, on the cytoplasmic domain of PTH1R can be directly phosphorylated by the IGF1R tyrosine kinase. The Y494-phosphorylation was induced by IGF1R at the cell surface. Disruption of the Y494-phosphorylation resulted in decreased actin polymerization and dendrite length.

We observed decreased PTH1R on dendritic protrusions with disruption of Y494-phosphorylation site relative to wild-type PTH1R, suggesting that the phosphorylation may serve as a sorting or packaging signal for the dispatch of PTH1R to its destiny on the actin tips. The mechanism by which P_Y_-PTH1R is transported from the cell membrane to the cytoskeleton is not yet fully understood. It is possible that alternative downstream signaling pathways other than PTH/cAMP or IGF-1/Akt may be involved. We also observed a preferential expression of PTH1R in osteocyte dendrites oriented towards the bone surface, implying a potential functional role of PTH1R on tips of dendrites, which warrants further investigation. Current knowledge indicates that the dynamic actin cytoskeleton plays an important role in clathrin-mediated endocytosis of member receptors^53,54^. The actin network can drive the departure of endocytosed vesicles away from the cell membrane and the transport of the vesicles into intracellular compartments. At the same time the membrane receptors in the vesicles can regulate structural dynamics of the actin network. The role of axon extension in neurons, including the polymerization of the barbed ends of actin filaments generating a pushing force for the extension of the cytoplasmic processes^55,56^, provide insight into future directions to pursue this study.

It is well established that local IGF-1/IGF1R signaling is necessary for the full anabolic effect of PTH on bone^14, 18-21, 25, 26,57^. Mice with global deletion of IGF-1 are largely unresponsive to PTH^18,19^. Liver specific IGF-1 knockout mice maintain skeletal PTH responsiveness^58^, whereas osteoblast-specific deletion of IGF1R is not responsive to PTH^20^ in regards to osteoblast differentiation and markers of bone formation. The effects have largely been contributed to increased IGF-1 mRNA and protein expression after PTH treatment^21–25^, which is supported in that IGF-1 alone is sufficient to stimulate the differentiation of MSCs/preosteoblasts to osteoblasts^59,60^ and regulate the attachment of preosteoblasts onto the bone surface^9^. The effect of PTH and IGF-1 on osteoblast-to-osteocyte transition has not previously been reported. We found that deletion of either IGF1R in osteoblast precursors or PTH1R in mature osteoblasts in vivo resulted in a decreased number of osteocytes and shortened dendritic processes. in vivo studies found that early osteocytes with a point mutation resulting in tyrosine to phenylalanine transcription at amino acid position 494 (Y494F) reduced the length of dendrites. Generation of a site specific Y494F mouse model would be needed to confirm these findings in vivo. Additionally, considering that the dendritic trafficking of PTH1R was only partially suppressed in Osx-Cre/IGF1R^*flf*^ mice, other tyrosine receptor kinases may also be able to phosphorylate PTH1R and mediate these effects. Other tyrosine receptors reported in osteoblasts include FGF-2 and EGF receptors. Ablation of either FGF-2 receptor or EGF receptor is also known to attenuate actions of PTH on bone^61–63^, suggesting that tyrosine receptor kinases targeting Y494 may not be limited to IGF1R. Finally, whether IGF-1 phosphorylation of PTH1R impacts dendrite length of mature osteocytes and function also remains to be explored.

Our data suggest that the Y494 phosphorylation of PTH1R by IGF1R plays a role in the morphological transition of osteoblasts into osteocytes during bone remodeling. Our results provide new insight into the molecular basis and pathways allowing specific functions and regulation of PTH signaling during osteoblast differentiation. We found that PTH1R is a unique substrate for IGF1R, which phosphorylates it on a site related to its preferential targeting to barbed ends of actin filaments. The phosphorylation integrates PTH and IGF-1 signaling during osteoblast-to-osteocyte transition and directs the outgrowth of dendrites. Further studies elucidating the roles of the P_Y_-PTH1R and understanding how it mediates the transition of osteoblasts into osteocytes are needed to better understand the process of bone remodeling and the mechanism for PTH anabolic effects on bone.

## Materials and methods

### Plasmids and antibodies

The PTH1R-HA/pcDNA3.0 and IGF1R-Flag/pcDNA3.0 plasmids were cloned from human PTH1R and IGF1R. Zipper-YFP1/pcDNA3.1 and zipper-YFP2/pcDNA3.1 have been described previously^64^. PTH1R-HA was introduced into pMSCVneo retroviral expression vector (BD Biosciences, San Jose, CA) to generate pMSCV-PTH1R-HA. PTH1R-CFP was generated by inserting PTH1R into pECFP vector (Clontech Laboratories, Mountain View, CA). IGF1R-YFP1 and PTH1R-YFP2 were generated by replacing the zippers in zipper-YFP1 or zipper-YFP2. GST-tagged cytoplasmic domain of PTH1R (amino acids 461–593, cPTH1R) was generated by fusing GST at N-terminals using pGEX-KG prokaryotic gene fusion vector (Pharmacia Corp., Pfizer Inc., New York, NY). GST-tagged cytoplasmic domain of IGF1R (cIGF1R) was purchased (Cell Signaling Technology, Danvers, MA). The mutants PTH1R-Y494F-HA and pMSCV-PTH1R-Y494F-HA were generated by primer-mediated PCR mutagenesis and verified via DNA sequencing. Adeno-Cre and Adeno-Cre-GFP were also purchased (Vector Biolabs, Malvern, PA). Primary antibodies used for immunoblotting, immunoprecipitation, or immunostaining included rabbit P_Y_-PTH1R antibody (IF, 1:150) (generated in current study 580 μg.mL^-1^); Flag antibody M2 (IB, 1:2000) (Sigma-Aldrich, St. Louis, MO); mouse HA antibody 16B12 (IB, 1:2000) and rabbit PTH1R antibody PRB-640P (IF, 1:100) (Covance, Princeton, NJ); mouse IGF1R antibody (IB, 1:1000), Rabbit P-IGF1R antibody (IB, 1:400), mouse AKT antibody 40D4 (IB, 1:2000), and Rabbit P-Akt antibody (IB, 1:500) (Cell Signaling Technology, Danvers, MA); Mouse PTH1R antibody (IF, 1:100) (Santa Cruz Biotechnology, Inc., Santa Cruz, CA).

### Mice

*PTH1R*^flox/flox^ (generous gift from Henry Kronenberg)^64^, *Oc-Cre* (generous gift from Thomas Clemens)^31^, *IGF1R*^flox/flox^ and *Osx-Cre* (Jackson Laboratory) mouse lines were used in the current study. *Oc-Cre/PTH1R*^*f/f*^ mice were generated by crossing *PTH1R*^flox/flox^ mice with *Oc-Cre* (referred as *Oc-Cre/PTH1R*^+/+^) mice. To generate *Osx-Cre/IGF1R*^*f/f*^ mice, hemizygous *Osx-Cre*/*IGF1R*^+/+^ mice were crossed with *IGF1R*^flox/flox^ mice to generate heterozygous *IGF1R*^+^*/*^*f*^ offspring with or without a *Cre* allele, these mice were then crossed with *IGF1R*^flox/flox^ mice to bring out *Osx-Cre/IGF1R*^*f/f*^ genotype. Female *Osx-Cre/IGF1R*^+/+^ and *Osx-Cre/IGF1R*^*f/f*^ whereas both male and female *Oc-Cre/PTH1R*^+/+^ and *Oc-Cre/PTH1R*^*f/f*^ were used in our studies. The genotype of transgenic mice was determined by PCR analyses of genomic DNA using the primers as previously described^9,65^. The bone phenotypes of *Oc-Cre/PTH1R*^*f/f*^ and *Osx-Cre/IGF1R*^*f/f*^ mice have been reported previously^9,34^. For PTH effects, 4-week-old mice were randomly divided to receive either, PTH (40 μg·kg^-1^) (Bachem Bioscience Inc., Torrance, CA) or vehicle (equivalent volume of 1 mmol·L^-1^ acetic acid in sterile PBS) in a final volume of 100 μL given daily by subcutaneous injection for 4 weeks. Both *Oc-Cre/PTH1R*^*f/f*^ (12.4 g ± 0.7 g) and *Osx-Cre/IGF1R*^*f/f*^ (16.7 g ± 1.2 g) 4-week-old mice were smaller than their wild-type littermates (19.8 g ± 0.8 g). Mice were maintained in the Animal Facility of Johns Hopkins University School of Medicine, housed in gangs in sterile, ventilated cages (Allentown Caging Equipment, Allentown, NJ), allowed free access to water and standard mouse chow, on a 14/10 h light/dark cycle. No adverse events occurred in the experimental animals. The Animal Facility complies with the Animal Welfare Act and maintains appropriate policies and procedures to ensure humane care and use of animals. The animal protocols were reviewed and approved by the Institutional Animal Care and Use Committee of the Johns Hopkins University, Baltimore, MD, USA.

### Fluorescence staining, confocal imaging, and histomorphometric analysis

Cells plated on glass coverslips were washed with cold PBS, fixed with 4% paraformaldehyde for 10 min, and then carefully permeabilized with 0.2% Triton X-100 for 6 min. The cells were then blocked with 10% BSA and incubated with primary antibodies in 2% BSA at 4 °C overnight, followed by incubation with a secondary antibody for 1 h. The stained cells were mounted with Vectashield medium (Vector Laboratories, Burlingame, CA) for confocal imaging. For bone specimens, femurs were dissected from animals and fixed in 10% neutral buffered formalin for 2 days and decalcified in 10% EDTA (pH 7.4) for 2 weeks. The decalcified bones were submerged in 30% sucrose to adjust their osmotic pressure and frozen in OCT compound, then sectioned sagittally into 8-μm-thick slices. The bone slices were permeabilized and blocked by incubation in 3% BSA, 0.1% Triton-100, 0.1% NaN3 for 2 h and stained overnight at 4 °C with primary antibodies, Texas red-, or FTIC-conjugated Phalloidin to stain for filamentous actin (F-actin) (1:200, Molecular Probes, Eugene, OR). For P_Y_-PTH1R staining, bone specimens were fixed in formalin overnight and treated with 10% EDTA for 30 min, then directly submitted for frozen sectioning. Confocal imaging was performed with an LSM510 confocal laser scanning microscopy system (Carl Zeiss, Oberkochen, Germany) at Ross Confocal Facility, Johns Hopkins University. The images were taken with 0.3-μm step size and processed four times with Kalman averaging. Three-dimensional reconstruction and the analysis of osteocyte dendrite length were conducted using software IMARIS (Bitplane, Switzerland). Additionally, quantitative histomorphometric analysis was conducted at the trabecular bone in a blinded fashion with OsteoMeasure^XP^ software (OsteoMetrics, Inc., Decatur, GA). Four randomly selected visual fields per specimen, in three specimens per mouse in each group were measured.

### Dendritic trafficking of PTH1R

Confocal laser scanning images from 25-μm-thick calvarial bone slices were reconstructed by software IMARIS for estimating the number and length of dendrites. The number and length of dendrites was measured by first rotating the osteocyte into a position such that the dendrite of interest extended only in the *X*–*Y* plane using Surpass View mode of IMARIS. The number of dendrites was counted manually. The length of dendrite was measured using Polygon Type in IMARIS. The five longest dendrites (from tip to cell body) in each osteocyte in 25-μm-thick slices and 20–30 osteocytes for each bone samples were measured. The number of dendrites reaching to the bottom layer of bone surface was used to estimate the connectivity between osteocytes and bone surface. To analyze the punctuate signals of PTH1R/ P_Y_-PTH1R, confocal laser scanning images from cultured cells or 8-μm-thick trabecular bone slices were reconstructed. The number of punctuations was analyzed using Spot function in IMARIS. Background subtraction was turned on. Quality threshold was 15. For cultured cells, estimated diameter for spots was setup to 3 μm. For bone specimens, the diameter for spots was setup to 2 μm. Osteocytes in the depth of 20 μm from bone surface were counted to estimate the number of PTH1R punctuations. About 5–10 osteocytes in four randomly selected visual fields in three specimens per mouse were measured.

### Transfection and reagents

HEK293 and UMR106 cells were obtained from the American Type Culture Collection (ATCC, Manassas, VA) and maintained in Dulbecco’s modified eagle medium (DMEM) or α-minimal essential medium (α-MEM) supplemented with 10% fetal bovine serum (FBS; Invitrogen Corp., Carlsbad, CA). Green fluorescent protein (GFP)-expressing BMSCs were obtained from the Texas A&M Health Science Center College of Medicine Institute (College Station, TX) and maintained in Iscove’s modified Dulbecco’s medium (IMDM) supplemented with 10% fetal calf serum (FCS; Atlanta Biologicals, Lawrenceville, GA), HEK293-PTH1R-CFP cells were generated by stable expression of PTH1R-CFP with pMSCVneo retrovirus transduction. The transfection of DNA plasmids were performed with Lipofectamine reagent (Invitrogen Corp., Grand Island, NY). Human PTH^1–34^ (here termed PTH) was purchased from Bachem, Inc. (Torrance, CA). PTH1R small peptide (residues 477–506) was synthesized by Genemed Synthesis, Inc. (San Antonio, TX); 100 nmol·L^-1^ of PTH, 50 ng·mL^-1^ IGF-1, 10 μmol·L^-1^ cytochalasin D (Sigma-Aldrich Corp., St. Louis, MO), 1 μmol·L^-1^ PPP (EMD Millipore, Billerica, MA) were used for cell treatment.

### Statistical analysis

Cell culture experiments were run in triplicate. For mouse studies, five mice were analyzed per group. Statistical differences between the two groups of data were analyzed with Student’s *t* test. Multiple comparisons were analyzed by one-way ANOVA for wild-type cells exposed to vehicle, IGF-1 and/or PTH and two-way ANOVA for wild type or knockout cells/mice exposed to either vehicle, IGF-1, or PTH with post-hoc Tukey procedure. The data are presented as mean ± s.d. All the data demonstrated a normal distribution and similar variation between groups. For all experiments, *P* < 0.05 were considered to be significant and indicated by *P < 0.01 were indicated by ‘**’. All inclusion/exclusion criteria were preestablished and no samples or animals were excluded from the analysis. The experiments were randomized. The investigators were not blinded to allocation during experiments and outcome assessment unless otherwise specified.

## Electronic supplementary material

Supplemental Information

Supplemental Figure 1
