# Supplementary material for: IGF-I induced phosphorylation of PTH receptor enhances osteoblast to osteocyte transition
Source: Bone Res. 2018 Feb 26;6:5. doi: 10.1038/s41413-017-0002-7 (PMC5827661; doi:10.1038/s41413-017-0002-7)
Supplement: Supplementary file 1 — Supplemental Information [file 41413_2017_2_MOESM1_ESM.docx]

**Supplemental Information**

**Supplemental Methods**

**Immunoprecipitation, immunoblotting, and fluorescence intensity analysis**

The cells were lysed in IP buffer (50 mM Tris-HCl pH 7.5, 150 mM NaCl, 1% Triton X-100, 0.5% sodium deoxycholate) containing protease inhibitors. The lysates were immunoprecipitated by incubation with the appropriate antibodies, followed by absorption on Protein G-Sepharose. The immunoprecipitates were separated by SDS–PAGE and blotted onto a nitrocellulose membrane. Immunoblots were characterized by using the SuperSignal West Femto Substrate system (Pierce, Thermo Fisher Scientific, Inc., Rockford, IL). Fluorescence intensity analysis for

YFP-PCA assay was performed as described previously (48). The results shown are means ± s.d. from four independent experiments.

**Fusion protein expression and kinase assay**

The GST-tagged cytoplasmic domains of PTH1R (residues 461–593) and GST-tagged cIGF1R were expressed in BL21 (DE3) cells, then pulled down by glutathione-agarose beads and added into kinase reaction that contained 50 mM Tris-HCl pH 7.5, 10 mM MgCl2 and 200 mCi of [γ-32P]ATP. The products were analyzed by 10% SDS–PAGE and the protein associated radiolabel was determined by the Storm 860 scanner. Mass spectrometric analysis was performed at the Prometic Core Facility of Johns Hopkins University.

**Primary cells, adenovirus infection and PCR**

Calvarial osteoblasts were isolated as described previously (50). To investigate the endogenous protein-protein interaction, calvarial osteoblasts were pre-cultured in osteogenic medium (0.1 M dexamethasome, 10 M glycerol phosphate, 0.05 M L-ascorbic acid in α-MEM) with 5% FBS for 3 days, then treated with IGF-1 or PTH. The BMSCs were collected from 4-week-old wild-type mice euthanized by cervical dislocation and cultured in α-MEM (Cellgro, Tewksbury, MA) supplemented 10% FBS (FBS, Atlanta Biologicals, GA). After 72 h of adhesion, we removed non-adherent cells and cultured adherent cells for an additional 7 d with a single media change. The adherent cells were then retrieved by 25% trypsin for 1-2 minutes. The easily detached cells were removed by washing with the medium. The cells still adhesive to the culture dish were treated again with 25% trypsin for 1-3 min and suspended in the medium. The cells were diluted and seeded at 100 cells/100 mm dish. After three-week culture, cells from different clones were collected and enriched by further culture, and then were screened using phycoerythrin (PE)-, peridinin chlorophyll protein (Per CP)- and allophycocyanin (APC)-conjugated antibodies to mouse Sca-1, CD45 and CD11b (BioLegend, San Diego, CA). Acquisition was performed on a fluorescence-activated cell sorting (FACS) Aria model (BD Biosciences, San Jose, CA), and the analysis was performed using FACS DIVE software version 6.1.3. The sorted Sca1^+^CD45^−^CD11b^−^ clone was cultured on bone slices of bovine cortical bone in osteogenic medium. At indicated times, the cells were suspended and seeded on glass coverslips for confocal imaging. For pMSCVneoretrovirus transduction, the cells were infected with pMSCV, pMSCV-PTH1R-HA, or the mutant pMSCV-PTH1R-Y494F-HA viruses for 4 h, and then cultured in α-MEM for 48 h. Stable transductants were selected by G418 for 1 week, followed by culture on bone slices in osteogenic medium. For PCR, total RNA was isolated using TRIzol reagent (Invitrogen, Grand Island, NY). cDNA synthesis was performed with TaqMan Reverse

Transcription reagent (Applied Biosystems, Foster, CA). PCR was performed using the primer sets: DMP1 (5’-GGCTGTCCTGTGCTCTCCCAG-3’) and (5’-

GGTCACTATTTGCCTGTGCCTC-3’); Phex (5’-GTGCATCTACCAACCAGATACG-3’) and

(5’-TCTGTTCCCCAAAAGAAAGG-3’); Sost (5’-CTTCAGGAATGATGCCACAGAGGT-3’) and (5’-ATCTTTGGCGTCATAGGGATGGTG-3’); GAPDH (5’-

AGGTCGGTGTGAACGGATTTG-3’) and (5’-GGGGTCG TTGATGGCAACA-3’).

**cAMP assay**

cAMP binding to Epac induces a conformational change that liberates the catalytic domain of Epac from intrasubunit allosteric inhibition. A chimeric protein (ICUE3) was generated by fusing the N terminus of a truncated Epac to ECFP and the C terminus to citrine, an improved version of YFP (21). Sandwiching such Epac-based conformationally responsive elements between a FRET pair allows monitoring of cAMP production and degradation by changes in

FRET. Therefore, changes in cAMP (changes in the ratio of cyan-to-yellow emissions) can be monitored in real time in living cells. In brief, HEK293 or UMR106 cells were co-transfected with ICUE3 and PTH1R-HA or PTH1R-Y494F-HA plasmids for 24 hours, and visualized under Olympus 1X80 microscope loaded with METAFLUOR 6.2 software (Universal Imaging). PTH induced changes in the ratio of cyan-to-yellow emissions were recorded and calculated as previously described (21).

**Alkaline phosphatase (ALP) activity and mineralization assays**

BMSCs were placed in 12-well plates at a density of 60% confluence and cultured in α-MEM medium for 2 days. Osteoblast differentiation was induced by the addition of osteogenic medium in low serum conditions (2% FBS) with supplement of PTH or IGF-1 or both. Chemical staining for ALP activity in the cells was performed at day 5 using Fast BCIP/NBT Tablets (Sigma-

Aldrich, St. Louis, MO). Alizarin Red staining for calcium deposits was performed at day 14.

Stained Alizarin Red was recovered by acetic acid extraction and neutralized with ammonium hydroxide, followed by colorimetric detection at 405 nm.
