# Supplementary figures and images for: IGF-I induced phosphorylation of PTH receptor enhances osteoblast to osteocyte transition
Source: Bone Res. 2018 Feb 26;6:5. doi: 10.1038/s41413-017-0002-7 (PMC5827661; doi:10.1038/s41413-017-0002-7)

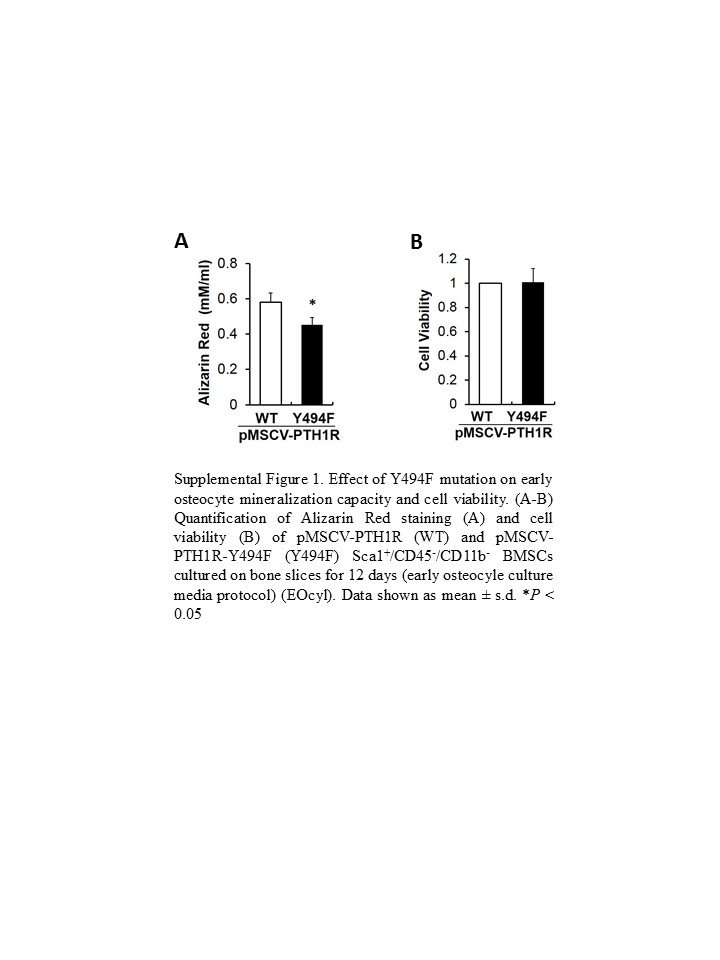

Supplement: Supplementary file 2 — Supplemental Figure 1 [file 41413_2017_2_MOESM2_ESM.jpg]
